# Impact of the Film Array Meningitis/Encephalitis panel in adults with meningitis and encephalitis in Colombia

**DOI:** 10.1017/S0950268820001648

**Published:** 2020-07-27

**Authors:** Karen Melissa Ordóñez Díaz, John Alexander Alzate Piedrahíta, Oscar Felipe Suárez Brochero, Daniel Orozco Granada, Laura Marcela Barón, Isabella Cortés Bonilla, Rodrigo Hasbun

**Affiliations:** 1 Hospital Universitario San Jorge, Pereira, Colombia; 2 Universidad Tecnológica de Pereira, Pereira, Colombia; 3 UT Health McGovern Medical School, Houston, TX, USA

**Keywords:** Antibiotic utilisation, Colombia, encephalitis, meningitis, meningitis–encephalitis multiplex PCR

## Abstract

The Biofire® Film Array Meningitis Encephalitis (FAME) panel can rapidly diagnose common aetiologies but its impact in Colombia is unknown. A retrospective study of adults with CNS infections in one tertiary hospital in Colombia. The cohort was divided into two time periods: before and after the implementation of the Biofire® FAME panel in May 2016. A total of 98 patients were enrolled, 52 and 46 were enrolled in the Standard of Care (SOC) group and in the FAME group, respectively. The most common comorbidity was human immunodeficiency virus infection (47.4%). The median time to a change in therapy was significantly shorter in the FAME group than in the SOC group (3 *vs.* 137.3 h, *P* < 0.001). This difference was driven by the timing to appropriate therapy (2.1 *vs.* 195 h, *P* < 0.001) by identifying viral aetiologies. Overall outcomes and length of stay were no different between both groups (*P* > 0.2). The FAME panel detected six aetiologies that had negative cultures but missed identifying one patient with *Cryptococcus neoformans*. The introduction of the Biofire FAME panel in Colombia has facilitated the identification of viral pathogens and has significantly reduced the time to the adjustment of empirical antimicrobial therapy.

## Introduction

Meningitis and encephalitis continue to be associated with significant neurological morbidity and mortality [1], so establishing a cause and administering prompt antimicrobial therapy is crucial in improving clinical outcomes in several aetiologies [2, 3]. Unfortunately, rapidly establishing an aetiological diagnosis in public hospitals in Colombia is not easy, given that most of these facilities do not have inhouse diagnostic tools such as specific polymerase chain reaction (PCR) for viruses and bacteria or latex for the detection of the capsular antigen of *Cryptococcus* spp. The performance of molecular diagnostic tests requires the shipping of the sample to a reference laboratory in either Medellin or Bogota with an approximate delay of 10–14 days to receive the results. Therefore, the possibilities of determining the causal agent were subject to the yield of traditional cultures, interpretation of the cerebrospinal fluid (CSF) cytochemistry and clinical findings prompting empirical therapy for the majority of patients [4]. In patients with bacterial meningitis, the sensitivity of the CSF Gram stain ranges from 10% to 93% and the CSF cultures between 50% and 85% depending on the pathogen, country of the study and by the receipt of previous antibiotic therapy [5]. This diagnostic uncertainty and the fear of an adverse outcome lead to an indiscriminate use of antimicrobials in the majority of patients. Clinicians prefer to initiate and maintain broad-spectrum therapies that include antibiotics, antivirals and in the case of HIV-positive antifungal patients, which increases not only treatment costs but also the risk of adverse events such as nephrotoxicity and *Clostridium difficile* diarrhoea [6].

The Biofire® Film Array Meningitis Encephalitis (FAME) panel is a multiplex PCR tool that utilises a sample of 200 μl of CSF to identify in 1 h the presence of 14 pathogens (*Escherichia coli K1*, *Haemophilus influenzae*, *Listeria monocytogenes*, *Neisseria meningitidis*, *Streptococcus agalactiae*, *S. pneumoniae*, *cytomegalovirus (CMV)*, *enterovirus (EV)*, *herpes simplex virus 1 (HSV-1)*, *herpes simplex virus 2 (HSV-2)*, *human herpes virus 6 (HHSV-6)*, *human parechovirus (HPeV)*, *varicella zoster (VZV)*, *Cryptococcus neoformans*/*C. gattii*) that was approved by the Federal Drug Administration (FDA) since 2015. Diagnostic correlation studies with CSF sample banks positive for the targets identified by the test have an agreement of >90% [7, 8]. The main limitation of this panel is with *C. neoformans* where it can be as low as 50% [8]. So far, studies have been carried out mainly in the pediatric population and in immunocompetent patients with overall good performance, although only approximately 25% of the film array panels are positive [9, 10]. Currently, there are no studies evaluating the performance of this panel in Latin America.

The Hospital San Jorge de Pereira is a tertiary care public hospital in the central-western region of Colombia, where the prevalence of HIV disease is one of the highest in the country. One of the most challenging infections is meningitis and encephalitis as the diagnosis is unknown in the majority of patients. In 2016, the Biofire® FAME panel was introduced. The aim of the present study was to evaluate the clinical impact of the introduction of this test in adults with meningitis and encephalitis.

## Materials and methods

The study was divided into two cohorts of adult patients with meningitis or encephalitis: before (Standard of Care (SOC) group) and after the introduction of the Biofire® FAME panel (FAME group). The inclusion criteria include adults (age >17 years) with a diagnostic suspicion of neuroinfection who had a CSF analysis and had complete medical records. Only the first episode of infection was taken into account. Data were collected through an electronic format which include demographic variables (age, sex), clinical (comorbidities), paraclinical (CSF cytochemical), culture results, FAME panel test result, days of antimicrobial treatment, difference in hours to make adjustments to the antimicrobial with microbiological test results (timeframe between the lumbar puncture to escalation, de-escalation or discontinuation of antimicrobial therapy), days were evaluated of total hospitalisation, days of hospitalisation in ICU, state of discharge (alive or dead). The cost of the diagnostic studies was calculated for both groups according to the protocol for the study of patients with clinical suspicion of central nervous system infection. The SOC for meningitis diagnosis included blood cultures, computed tomography scans with magnetic resonance imaging testing when encephalitis was suspected. The study of CSF included culture, Gram stain, India ink stain, fungal culture. If the patient presented HIV diagnosis, the study of CSF included CMV PCR and cryptococcal antigen test.

FAME studies included blood cultures, computed tomography scans, magnetic resonance imaging testing when encephalitis was suspected. The study of CSF included culture, Gram stain, India ink stain, Biofire® FAME panel. HIV and non-HIV patients were studied in the same way.

According to the protocol at the hospital, patients with clinical suspicion of neuroinfection were assessed by neurology and infectious diseases. Antimicrobial treatment was initiated according to guidelines (ceftriaxone and vancomycin for bacterial meningitis, amphotericin B-antituberculous-ceftriaxone-ampicillin for patients with HIV disease, and acyclovir for suspected encephalitis) and a CSF study was performed to perform diagnostic tests. The FAME panel was requested by either the neurologist or the infectious diseases specialist, the latter one interpreted the results and adjusted the medical management as soon as the panel was done. An appropriate antimicrobial therapy was defined as a therapy initiated with a diagnosis of meningitis/encephalitis constructed by clinical signs and symptoms, laboratory findings compatible with infection, with adjustment according to Gram stain results, culture results and PCR results when those were available. The antimicrobial stewardship programme headed by an infectious diseases specialist determined the concept of appropriate therapy.

The study was approved by the Institutional Review Board of the Hospital San Jorge de Pereira.

### Statistical analysis

The results were described as frequencies and medians with interquartile ranges between FAME and SOC groups. Statistical differences were assessed by *χ*^2^ and Fisher's exact test, when comparing categorical variables, and by Mann–Whitney *U* test for comparing medians. Main outcomes were time to appropriate therapy, length of stay and in-hospital mortality. Glasgow Outcome Scale (GOS), use of diagnostic methods (MRI, head CT scan, blood culture bottles) and use of antimicrobials were secondary outcomes.

## Results

Of 118 patients reviewed, 20 patients were excluded because a neuroinfection was ruled out. A total of 98 patients met the inclusion criteria for the study. Of these, 52 (52.5%) patients were in the SOC group before the implementation of the panel and 46 (47.5%) patients were in the FAME group. The demographic characteristics and outcomes between the two groups of patients are shown in Table 1. There were no differences in regards to sex, clinical characteristics, comorbidities and outcomes (*P* > 0.05) but patients in the FAME group were older (*P* = 0.04).

**Table 1.** Comparison of baseline characteristics and outcomes between Standard of Care (SOC) and Film Array Meningitis-Encephalitis (FAME) groups

Baseline variables	SOC (*n* = 52)	FAME (*n* = 46)	*P* value
Median age (years, range)	36 (25.5–45.5)	43 (34–55)	0.040†
Female sex, *n* (%)	8 (15.4)	14 (30.4)	0.075
Clinical characteristics, n (%)			
Headache	40 (76.9)	29 (63)	0.133
Altered mental status	28 (53.8)	29 (63)	0.357
Meningeal signs	27 (51.9)	23 (50)	0.849
Seizures	22 (42.3)	17 (36.9)	0.589
Focal neurological exam	6 (11.5)	7 (15.2)	0.592
Charlson comorbidity score (median, IQR)	6 (IQR 1- 7)	6 (IQR 1–7)	0.956 †
Human immunodeficiency virus (HIV positive), *n* (%)	25 (48)	23 (50)	0.849
Comorbidities, *n* (%)			
Diabetes mellitus	3 (5.7)	4 (8.7)	0.575
Chronic renal failure (stage IV–V)	1 (1.9)	3 (6.5)	0.251
Tuberculosis	5 (9.6)	5 (10.8)	0.930
Solid tumours	1 (1.9)	4 (8.7)	0.128
Neutropenia	1 (1.9)	0	-

IQR, interquartile range.

As shown in Table 2, there were no differences in the CSF profile between the SOC and FAME groups (*P* > 0.05). A positive CSF India ink and Gram stain were seen in 5% and 12% of patients, respectively, with no differences between the two groups (*P* > 02). A positive CSF culture was seen more frequently in the SOC group (21% *vs* 6.5%, *P* = 0.039).

**Table 2.** Cerebrospinal fluid (CSF) results and aetiologies between Standard of Care (SOC) and Film Array Meningitis-Encephalitis (FAME) groups

Variables	SOC (*n* = 52)	FAME (*n* = 46)	*P* value
Median CSF WBC (cell/ml)	12 (1–69)	21.5 (0–152)	0.441
Median neutrophil per cent (%, IQR)	0 (0–25)	10 (0–30)	0.201
Median CSF protein (g/dl, IQR)	104 (29–228)	97 (46–223)	0.363
Median CSF glucose (m/dl, IQR)	48 (30–60)	43 (30–54)	0.385
Positive Gram stain, *n* (%)	8 (15.4)	4 (8.7)	0.313
Positive Indian Ink, *n* (%)	4 (7.7)	1 (2.1)	0.735
Positive CSF culture, *n* (%)	11 (21.1)	3 (6.5)	0.039
Aetiologies, *n* (%)	14^a^ (26.9)	17 (36.9)	0.286
Bacterial meningitis	7^b^	5^c^	
*Cryptococcus neoformans*	7	6	
Cytomegalovirus	0	3	
Herpes simplex virus	0	3^d^	

WBC, white blood cell; IQR, interquartile range; mg, milligram; dl, decilitre; g, gram.

a In SOC group, the isolation of *E. coli* was in blood cultures, 1 diagnosis of *C. neoformans* was by latex antigen (negative cultures), 6 positive CSF cultures for *C. neoformans*, the other bacterial isolates were identified in CSF culture only.

b *S. pneumoniae* (2), *K. pneumoniae* (1), *E. coli* (1). Gram-positive bacilli (1), Gram-positive cocci (1), Gram-negative cocci (1).

c *S. pneumoniae* (2), *N. meningitidis* (1), *H. influenzae* (1), Gram-positive cocci (1).

d *Herpes simplex virus* type 1 (1), *Herpes simplex virus* type 2 (2).

As shown in Table 2, 14 (27%) of the SOC had an aetiology identified and 17 (36.9%) in the FAME group. Only the FAME group identified viral aetiologies (three with cytomegalovirus and three with herpes simplex viruses). All other aetiologies in both groups were either bacterial or fungal. As shown in Table 3, only two out of four positive FAME panels for bacteria were also detected by cultures. In regards to *C. neoformans*: four positive FAME had negative CSF cultures, one patient had both a positive FAME and culture and one patient had a negative FAME but a positive CSF culture for *C. neoformans*.

**Table 3.** Concordance of the Biofire Film Array Meningitis Encephalitis (FAME) with blood and cerebrospinal fluid (CSF) cultures

Aetiology	FAME result	Culture results
*Streptococcus pneumoniae*	*S. pneumoniae*	*S. pneumoniae* on CSF only
*Streptococcus pneumoniae*	*S. pneumoniae*	Negative on blood and CSF
*Neisseria meningitidis*	*N. meningitidis*	*N. meningitidis* isolated only in blood culture
*Haemophilus influenzae*	*H. influenzae*	Negative blood and CSF
Gram-positive cocci (Gram stain only)	Negative	Negative blood and CSF
*Cryptococcus neoformans*	Negative	*C. neoformans* isolated in CSF only
*Cryptococcus neoformans*	*C. neoformans*	*C. neoformans* isolated in CSF only
*Cryptococcus neoformans* (4)	*C. neoformans*	Negative blood and CSF

Overall outcomes and length of stay in the hospital or in the intensive care unit were no different between both groups (*P* > 0.2) (see Table 4). The median time to a change in therapy was significantly shorter in the FAME group than in the SOC group (3 *vs.* 137.3 h, *P* < 0.001). This difference was driven by the timing to appropriate therapy (2.1 *vs.* 195 h, *P* < 0.001) mostly driven by identifying viral aetiologies such as CMV and HSV in a timely fashion (Table 5). The prolonged delay initiation in the SOC group was seen mainly in non-HIV patients with cryptococcal meningitis, and in some patients with viral encephalitis not suspected by clinical presentation or CSF characteristics.

**Table 4.** Lengths of stay, mortality, time to appropriate therapy and diagnostic work up between Standard of Care (SOC) and Film Array Meningitis Encephalitis (FAME) groups

Variables	SOC (*n* = 52)	FAME (*n* = 46)	*P* value
Median in hospital length of stay (days) (IQR)	18 (7.5–28)	17 (12–27)	0.786
Median ICU length of stay (days) (IQR)	4 (1–11)	6 (3–10)	0.502
Glasgow Outcome Score^a^	3 (1–4)	4 (1–4)	0.214
In hospital mortality, *n* (%)	17 (32.7)	15 (32.6)	0.993
Median time from test result to any therapy change (initiation or discontinuation) (hours, range)	137.3 (46–195)	3 (1.8–20.3)	⩽0.001
Time from test result to initiation of appropriate therapy	195.4 (146.7–234.7)	2.1 (1.6–4.6)	⩽0.001
Time from test result to discontinuation of inappropriate therapy	92 (47–190)	19.1 (2–71)	0.050
Blood culture bottles (>5)	14 (26.9)	5 (10.8)	0.045
Head CT scan (>2)	8 (15.3)	3 (6.5)	0.165
MRI of the brain	11 (21.1)	19 (41.3)	0.031

IQR, interquartile range; ICU, intensive care unit; CT, computed tomography; MRI, magnetic resonance image.

a Glasgow outcome score: a score of 1 indicates death, a score of 2 indicates a vegetative state (inability to interact with the environment), a score of 3 indicates severe disability (unable to live independently but follows commands), a score of 4 indicates moderate disability (unable to return to work or school but able to live independently), and a score of 5 indicates mild or no disability (able to return to work or school).

**Table 5.** Time (hours) to change in therapy between Standard of Care (SOC) and Film Array Meningitis-Encephalitis (FAME) groups according to microbiologic results

Time (hours) to change in therapy (median, IQR)	SOC	FAME	*P* value
If use of any antibiotic (12 *vs*. 12)	137 (46.4–192)	2.5 (1.5–13.1)	⩽0.001
If use of any antiviral (5 *vs*. 9)	146.7 (48.5–173)	2 (1.3–19.3)	0.013
If use of Amphotericin B (5 *vs*. 6)	195 (92–217)	2 (1.2–6.1)	0.010
If neuroinfection confirmed (13 *vs*. 20)	127.8 (47–190)	2.4 (1.8–24.7)	⩽0.001
If neuroinfection discarded (1 *vs*. 4)	217	5 (3–13.3)	0.157

IQR, interquartile range.

Furthermore, there was a trend that inadequate antimicrobial therapy was discontinued in a timelier manner in the FAME group (19.1 *vs.* 92 h, *P* = 0.05). Patients in the SOC group also had more blood cultures done than in those in the FAME group (*P* = 0.045). Patients in the FAME group had more magnetic resonance imaging of the brain, a finding associated with the acquisition of the resource by the institution during this period. As shown in Table 6, there were no differences in the use and duration of the different antimicrobial therapies between both groups (*P* > 0.5). The median cost of antimicrobial treatment in the SOC group was US$456.48 (US$31.01–US$854.56) per patient treatment course compared to the median cost of antimicrobial treatment in FAME group that was US$309.81 (US$0–US$694.74) per patient (*P* = 0.184). When including the cost of diagnostic testing, the median cost per patient using the SOC was US$755.4 (US$251.58–US$1128.82) per treatment course *vs.* US$602.09 (US$398.08–US$1071.34) in the FAME group (*P* 0.685).

**Table 6.** Antimicrobial use and duration between Standard of Care (SOC) and Film Array Meningitis-Encephalitis (FAME) groups

Antimicrobial therapy use (*n*, %) and duration in days (median, IQR)	SOC (*n* = 52)	FAME (*n* = 46)	*P* value
Use of any antibiotic	32 (61.5)	20 (42.5)	0.059
Duration of all antibiotics	5 (2–8.9)	6.5 (1–10.7)	0.72
Ampicillin	9 (17.3)	7 (14.89)	0.745
Duration	1.5 (0–6)	4 (0.5–8)	0.296
Ceftriaxone	23 (44.2)	15	0.239
Duration	5 (1–8)	(32.6)	0.787
Cefepime	8 (15.4)	6 (0–9)	0.067
Duration	8 (1–10)	2 (4.2)	1.000
Meropenem	8 (15.4)	10 (0–20)	0.067
Duration	1 (0–3.5)	2 (4.2)	0.891
Vancomycin	23 (44.2)	1 (0–2)	0.087
Duration	6 (1–10)	13 (27.6)	0.767
Use of any antiviral	14 (26.9)	5 (1–8)	0.332
Duration of all antiviral	6.5 (4.5–11)	12(25.5)	0.875
Acyclovir,	13 (25)	11.5 (3–15)	0.401
Duration	5.5 (3–8)	8 (17.0)	0.524
Gancyclovir	1 (1.9)	5 (3–12)	0.135
Duration	0 (0–0)	4 (8.5)	0.124
Amphotericin B	16 (30.77)	16 (0–20)	0.184
Duration	17 (12–22)	9 (19.1)	0.067
Antituberculous therapy	9 (17.3)	12.5 (5–14) 14 (29.8)	0.142

IQR, interquartile range.

## Discussion

The present study was conducted to evaluate the clinical impact of the implementation of the FAME panel as part of the protocol for the study and management of adult patients with meningitis or encephalitis in a public hospital in Colombia. The proportion of positive results of the FAME panel in this study was higher (34.7%) than seen in other previous studies (8.7% [7]; 10.4% [11]; 12.7% [9]).

We had no false-positive FAME results in our study. All of the positive results had either a consistent clinical and CSF picture or an isolation in culture (for fungi and bacteria). No additional PCR tests were performed to confirm the viral aetiologies. Unlike other studies done in the USA where the main aetiologies were viruses, the main aetiological agent in our study before and after the establishment of the panel was *C. neoformans*. This finding is related to the high prevalence of HIV disease in the population studied (~50%).

Only patients in the FAME group were able to identify viral aetiologies such as CMV and HSV. Furthermore, the FAME panel was able to detect bacteria and *C. neoformans* in patients with negative CSF and blood cultures. This could be in some patients due to previous antibiotic therapy. We also observed a false-negative result in a patient with culture-confirmed cryptococcal meningitis. The presence of false negatives in cryptococcal meningitis in patients studied with the ME panel coincides with recent literature [8, 12], but differs from that found in the studies conducted in Uganda where a high concordance rate was seen with culture and latex antigen [13]. Given the severity of the outcomes that may result from stopping an antifungal therapy due to a false-negative FAME panel, it is recommended that if the clinical suspicion of cryptococcosis is high not to discontinue antifungal therapy until a CSF cryptococcal latex antigen is obtained or the CSF culture is finalised [8]. The panel does not identify Epstein–Barr Virus (EBV), which has been related with neuronal inflammation, endothelial damage (even in the absence of clinical encephalitis) and central nervous system lymphoma in immunosuppressed patients, with impact in the development of cognitive dysfunction and psychiatric symptoms [14]. EBV is also associated with post-transplant lymphoproliferative disorder and occasionally with encephalitis in solid organ transplant patients' recipients [15].

In the SOC period, the aetiological diagnosis depended on the result of CSF culture, blood cultures as well as the PCR of viruses in CSF. The usual time to obtain cultures ranged between 3 and 5 days and that of the PCR between 14 and 21 days to be processed in reference laboratories outside of Pereira. During the FAME period, there was a significant improvement in the time to obtain the results that were associated with changes in empirical antimicrobial, both initiating appropriate therapy and discontinuing inappropriate therapy. The difference in costs of including the Biofire® FAME panel was not significant compared to SOC. There was a tendency of reduction in the cost of antimicrobial therapy and the cost of diagnostic studies with the implementation of the Biofire® FAME panel. To our knowledge, this is the first study documenting this impact of the FAME panel in Colombia. This also resulted in the practice of repeating blood cultures in the FAME period. A recent study evaluated the cost-effectiveness of setting up the ME panel, it was found that the main benefit from the economic point of view was in the decrease in the consumption of antimicrobials [16]. This finding contrasts with another study [10] where there was no timely suspension of inappropriate therapy according to the results of the panel, which reinforces the importance of the implementation of the test in the context of an antimicrobial stewardship programme.

Given the costs of the test, it is currently unclear if all CSF samples with suspected neuroinfection or only those with CSF pleocytosis should undergo testing with the panel. A paediatric study showed that EV may present without pleocytosis in infants suggesting that it may be cost-effective in a universal approach with the ME panel by reducing the number of studies and days of hospitalisation [17].

Limitations of the study included the limited number of patients who underwent the test, the completion of the study in a single centre and the non-confirmation of the positive results of the virus with another test. Furthermore, the sample size also did not allow a differential analysis of results by type of aetiology, which may show differences with respect to the global analysis and possibly was associated with a low power in detecting an impact on clinical outcomes.

As for strengths, this study is the first to evaluate the clinical impact of the implementation of the Biofire® FAME panel test in Colombia as part of the study plan of adult patients with clinical suspicion of neuroinfection. Given the results presented, it is considered that the test is useful in the study of patients with neuroinfection mainly in the reduction of antimicrobial consumption in the context of an antibiotic control programme and directed according to alterations of the CSF cytochemistry.

In conclusion, the implementation of the FAME panel in a public hospital in Colombia resulted in a more rapid diagnosis that improved the detection of pathogens and had an impact on appropriately modifying the empirical antimicrobial management of patients but had no impact of lengths of stay or outcomes. Future studies should validate these results in other Latin American countries.

## Data Availability

The data for the study are available by contacting the corresponding author.
